# Chionanthus Virginica

**Published:** 1886-07-20

**Authors:** John A. Henning

**Affiliations:** Garnett, Kansas


### CHIONANTHUS VIRGINICA.

By John A. Henning, M. D., Garnett, Kansas.

This is a shrub or small tree growing along creeks or rivers, mostly in the Southern States. Leaves obovate, lower surface downy ; flowers, loose panicles in early summer ; petals about an inch long, and the fruit blue-purple, with a bloom ; common name, *fringe-tree.* The bark of the root is the only part employed. It is made and used in the form of a fluid extract. Dr. Justice has found saponine in it, but the plant has not come into general use.

Having used the fluid extract of chionanthus extensively in my practice for the last ten years, I can speak from personal experience as to its therapeutic action.

To simply say that a good article of the fluid extract, given in doses of half to one fluidrachm two or three times per day, is an aperient, cholagogue, diuretic, and an alterative, would but imperfectly describe its physiological effect in the human system. Of course we should and must know its therapeutic effect, but we also must know when and in what pathological condition the system is in before we can prescribe it rationally. Some writers have said heretofore that chionanthus is a specific in jaundice. Although I believe there are no specifics for any disease, my experience warrants me in saying that this remedy is one of the best in jaundice that I have ever prescribed. Chionanthus is indicated in all cases where there is a yellow, jaundiced condition of the skin. While it is but a feeble hepatic stimulant, I think it is one of the most reliable remedies in congestion of the portal circulation that we have. I have seen this verified in a large number of cases. It also seems to stimulate the lymphatic system, and to possess some diaphoretic and diuretic action.

If the liver is torpid, other hepatic stimulants should be used, such as podophyllin or leptandrin. The following prescription is the form in which I use this remedy:

℞. Ext. fld. chionanthin.......... ..................℥ i,
Ext. fl. podophyllum ..............................ʒ i,
Potass. acetat ..................................℥ ss,
Aquæ, q. s. ad...................................℥ iv.
M. Dose one teaspoonful three times per day.

In all jaundiced conditions this prescription has never failed me. However, other remedies may be indicated, as jaundice is but a symptom, not a disease, and many causes may produce it.—*Ther. Gazette.*

